# Hourly simulation results of building energy simulation tools using a reference office building as a case study

**DOI:** 10.1016/j.dib.2021.107370

**Published:** 2021-09-14

**Authors:** Mara Magni, Fabian Ochs, Samuel de Vries, Alessandro Maccarini, Ferdinand Sigg

**Affiliations:** a Unit for Energy Efficient Buildings, University of Innsbruck, Innsbruck Austria; b Eindhoven University of Technology, Eindhoven the Netherland; c Aalborg University Copenhagen Denmark; d Technische Hochschule Rosenheim Germany

**Keywords:** Building simulation, Cross comparison, Statistical indices

## Abstract

The data presented in this article are the results of widespread building simulation tools (i.e. EnergyPlus, TRNSYS, Simulink/CarnotUIBK, Simulink/ALMABuild, IDA ICE, Modelica/Dymola and DALEC) used to simulate a characteristic office cell, described within IEA SHC Task 56 [1], located in Stockholm, Stuttgart and Rome. Hourly data for each component of the thermal balance (i.e. Heating, cooling, infiltration, ventilation, internal gains, solar gains) and the hourly convective and radiative temperatures are reported for all the tools along with the ambient temperature and solar irradiation on the south façade. The mainly used statistical indices (i.e. Mean Bias Error, Mean Absolute Error, Root Mean Square Error and coefficient of determination) are applied to evaluate the accuracy of the tools. For more insight and interpretation of the results, please see “Detailed Cross Comparison of Building Energy Simulation Tools Results using a reference office building as a case study” [2]. This data set and evaluation methods are made available to ease the cross-validation process for other researchers.

## Specifications Table

**Table:** 

Subject	Engineering, Architecture
Specific subject area	Building energy simulation: tools cross-comparison (i.e. EnergyPlus, TRNSYS, Simulink/CarnotUIBK, Simulink/ALMABuild, IDA ICE, Modelica/Dymola and DALEC).
Type of data	Table, Graph, Text
How data were acquired	Output of building energy modeling – Computer simulation using the following software programs: EnergyPlus, TRNSYS, Modelica, IDA ICE, Simulink/CarnotUIBK, Simulink/ALMABuild, DALEC.
Data format	Raw Analysed
Parameters for data collection	The outputs of each building energy simulation tool, included in this comparison are used as a basis for the evaluation.
Description of data collection	Hourly data of each component of the energy balance (i.e. Heating, cooling, infiltration, ventilation, internal gains, solar gains) and convective and radiative temperature along with the ambient temperature and solar irradiation on the south façade as a result of the simulation of the reference office cell located in Rome, Stuttgart and Stockholm.
Data source location	The evaluations are performed considering the following climates: Rome-Fiumicino Country: Italy Latitude and longitude for collected samples/data: 41.80, 12.233 Stuttgart-Echerd Country: Germany Latitude and longitude for collected samples/data: 48.68, 9.22 Stockholm-Bromma Country: Sweden Latitude and longitude for collected samples/data: 59.35, 17.95
Data accessibility	With the article
Related research article	Magni M., Ochs F., de Vries S., Maccarini A., Sigg F., Detailed Cross Comparison of Building Energy Simulation Tools Results using a reference office building as a case study, Energy and Buildings, 250 (2021), https://doi.org/10.1016/j.enbuild.2021.111260.

## Value of the Data

• The hourly results of the cross-validated tools (i.e. EnergyPlus v.9.3, TRNSYS 18, Simulink/CarnotUIBK, Simulink/ALMABuild, IDA ICE v.4.8, Modelica Buildings library v.5.0.1 together with Dymola v. 2020x, DALEC) are reported for each component of the energy balance and for the convective and radiative temperature providing a wide dataset that can be used for the validation of other models for the simulation of office buildings.
• All the users of building simulation tools that would like to cross-compare their model and do not have available measurements can benefit from this dataset.
• The hourly results of a building simulation model can be cross-validated using this dataset as a reference, where the main used statistical indices are already calculated and can be used for a detailed evaluation of deviations.
• The proposed method for the evaluation of deviations between time series can be applied to the results of building simulations focusing on different building typologies. In addition, measured data, if available, can replace the median value that is used here as a reference, extending the usability of the proposed excel sheet to different case studies.

## 1 Data Description

The data set includes an excel file for each considered location (i.e. Rome, Stuttgart and Stockholm). Each spreadsheet includes ten tables (i.e. Heating, cooling, infiltration, ventilation, solar gains, internal gains, convective temperature, radiative temperature, ambient temperature and solar irradiation on the south façade) with the hourly results of each considered tool (i.e. EnergyPlus, TRNSYS, Simulink/CarnotUIBK, Simulink/ALMABuild, IDA ICE, Modelica, DALEC). The names of the tools will be abbreviated as follows and the abbreviations are used in the following sections and in the excel file:

• EP: EnergyPlus;
• TRN: TRNSYS;
• SIM IBK: Simulink/CarnotUIBK;
• SIM BO: Simulink/ALMABuild;
• IDA: IDA ICE;
• MOD: Modelica;
• DAL: DALEC.

In each, excel sheet the median of all the tools is calculated as well as the total annual energy or average temperature. Table 1 reports a section of the table reporting the heating powers for the climate of Stockholm. The first line of Table 1 shows the total energy and the last column reports the median of all the tools for each hour.

**Table 1:** Hourly heating power for the climate of Stockholm.

TOT [kWh/m^2^]	16.7	18.2	16.9	17.1	18.0	16.9	18.0	17.3
	Hourly average power [Wh/m^2^]							
Time / [h]	EP	TRN	SIM IBK	SIM BO	IDA	MOD	DAL	MEDIAN
**…**	…	…	…	…	…	…	…	…
**50**	5.2	7.6	6.8	7.2	8.1	5.7	0.0	6.8
**…**	…	…	…	…	…	…	…	…

On the right side of the hourly results, the statistical indices discussed and described in [2] and in Section 2.3 are calculated and reported as shown in Table 2. Here below the used acronyms are listed:

• MBE: Mean Bias Error;
• MAE: Mean Absolute Error;
• RMSE: Root Mean Square Error;
• NMBE: Normalized Mean Bieas Error;
• NMAE: Normalized Mean Absolute Error;
• NRMSE (av): Normalized Root Mean Square Error calculated using the average of the reference values as normalization means;
• NRMSE (|av|>0): Normalized Root Mean Square Error calculated using the average of the absolute reference values higher than zero as normalization means;
• R2: Coefficient of determination.

**Table 2:** Statistical indices.

	EP	TRN	SIM IBK	SIM BO	IDA	MOD	DAL
MBE [Wh/m^2^]	−0.07	0.11	−0.04	−0.03	0.08	−0.04	0.07
MAE [Wh/m^2^]	0.41	0.13	0.27	0.09	0.42	0.18	0.60
RMSE [Wh/m^2^]	1.59	0.35	0.61	0.26	0.95	0.45	1.37

NMBE [%]	−3.6	5.3	−2.3	−1.3	4.1	−2.3	3.7
NMAE [%]	0.0	6.5	13.8	4.5	21.1	9.3	30.2
NRMSE (av) [%]	80.2	17.5	31.1	13.1	48.3	22.8	69.5
NRMSE (|av|>0) [%]	24.1	5.3	9.3	3.9	14.5	6.8	20.9
R2 [%]	80	99	97	99	93	98	85

**Fig. 1:**
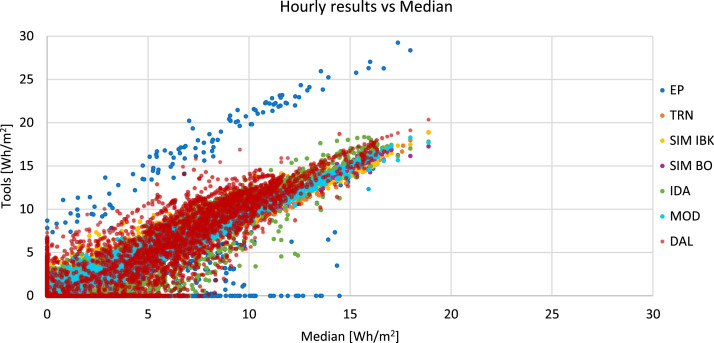
Hourly results (i.e. heating power for the climate of Stockholm) of each tool plotted against the median value.

To ease the visualization of the hourly results and calculated statistical indices the following graphs are placed in each excel sheet. Fig. 1 where the hourly results of each tool (i.e. in this case, the heating power for the climate of Stockholm is reported) are plotted against the reference results (i.e. median of all the tools). Here the spread of the results can be visualized.

**Fig. 2:**
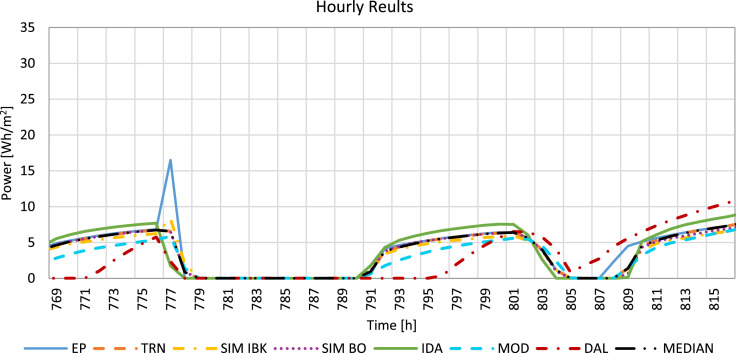
Hourly results (i.e. heating power for the climate of Stockholm) of each tool and of the median for a user-selected period.

In Fig. 2 the hourly results of each tool are reported along with the hourly median value for a time frame that can be selected by the user of the excel file.

**Fig. 3:**
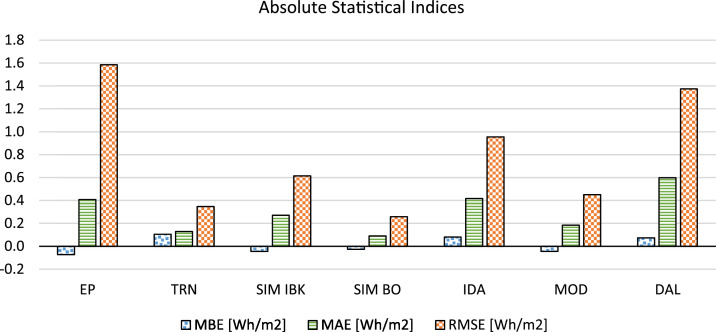
Absolute statistical indices (i.e. MBE: mean bias error, MAE: mean absolute error, RMSE: root mean square error).

In Fig. 3 the results of the absolute statistical indices (i.e. MBE: Mean Bias Error, MAE: Mean Absolute Error, RMSE: Root Mean Square Error) reported in Table 2 are presented.

**Fig. 4:**
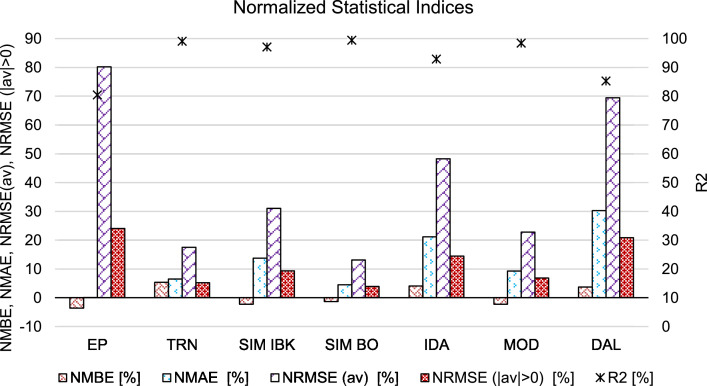
Normalized statistical indices (i.e. NMBE: Normalized Mean Bias Error, NMAE: Normalized Mean Absolute Error, NRMSE (av): Normalized Root Mean Square Error calculated using the average of the reference values as normalization means, NRMSE (|av|> 0): Normalized Root Mean Square Error calculated using the average of the absolute reference values higher than zero as normalization means, R2: coefficient of determination).

In Fig. 4 the results of the normalized statistical indices (i.e. NMBE: Normalized Mean Bias Error, NMAE: Normalized Mean Absolute Error, NRMSE (av): Normalized Root Mean Square Error calculated using the average of the reference values as normalization means, NRMSE (|av|>0): Normalized Root Mean Square Error calculated using the average of the absolute reference values higher than zero as normalization means, R2: coefficient of determination) reported in Table 2 are presented.

## 2 Experimental Design, Materials and Methods

Within the following sections, a short description of the office cell is provided, some key information about the applied tools and post-processing of the results are given and finally, the equations used for the analysis of the deviations between the dynamic results are provided. A detailed description of the methodology is also provided in [2].

### 2.1 Building and boundary conditions description

The reference office buildings described within IEA SHC Task 56 [1], located in Stockholm, Stuttgart and Rome is used within this work as it represents a characteristic office cell located on the middle floor of a high-rise building. The same Typical Meteorological Year (TMY2) for each location is used as input of the dynamic building simulation tools. Table 3 reports the yearly average ambient temperature ($\bar{\vartheta }$_amb,av_), global irradiation on a horizontal surface (I_g,hor_) and irradiation on a south-oriented vertical surface (I_south_) characterizing the weather in each considered location.

**Table 3:** Main boundary conditions: yearly average ambient temperature ($\bar{\vartheta }$_amb,av_), yearly global irradiation on a horizontal surface (I_g,hor_) and yearly irradiation on a south-oriented vertical surface (I_south_) [2].

Location	$\bar{\vartheta }$_amb,av_	I_g,hor_	I_south_
	[ °C]	[kWh/m^2^]	[kWh/m^2^]
Rome	15.8	1632	1253
Stuttgart	9.9	1101	889
Stockholm	7.8	952	884

**Fig. 5:**
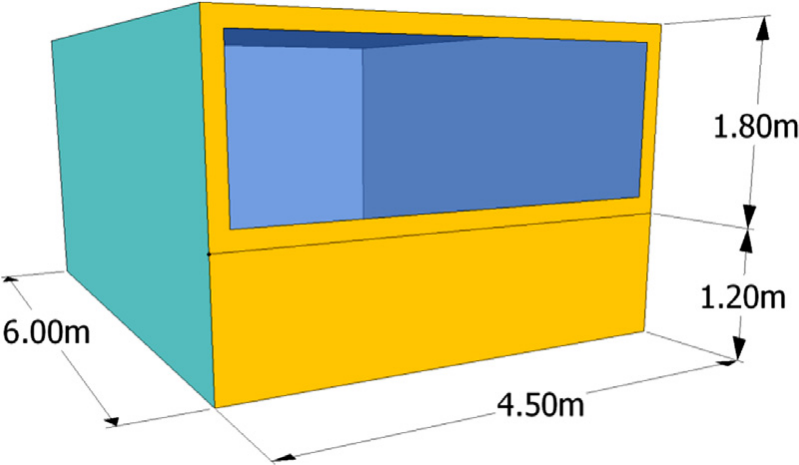
Representation of the reference office building [2].

The office has a heated area of 27 m^2^ and a volume of 81 m^3^ (see Fig. 5). The south-oriented façade disposes of a large window (i.e. window to wall ratio of 60%) and is the only one considered as non-adiabatic. A movable shading system, activated when the direct solar radiation impinging the south façade is higher than 120 W/m^2^ and able to block the 70% of the incoming solar radiation is considered within this case study to reduce overheating problems.

The heat transfer coefficient (HTC) of the opaque wall element and the characteristics of the windows such as HTC, Solar Heat Gain Coefficient (SHGC) and the solar transmittance (τ_sol_) for the three climates are listed in Table 4.

**Table 4:** Main properties of the south-oriented façade [2].

Properties	Rome (Italy)	Stuttgart (Germany)	Stockholm (Sweden)
HTC_ext,wall_ [W/(m^2^K)]	0.80	0.40	0.30
HTC_win_ [W/(m^2^K)]	1.26	1.35	0.90
SHGC [%]	0.33	0.59	0.63
τ_sol_ [%]	0.26	0.43	0.46

A constant air change rate of 0.15 ACH is assumed to account for natural infiltration while an additional airflow rate of 120 m^3^/h is supplied by a mechanical ventilation system with a sensible heat recovery efficiency of 70%. The heat recovery system is bypassed when free cooling is beneficial (i.e. air temperature of the thermal zone higher than 23 °C and higher than the ambient temperature).

**Fig. 6:**
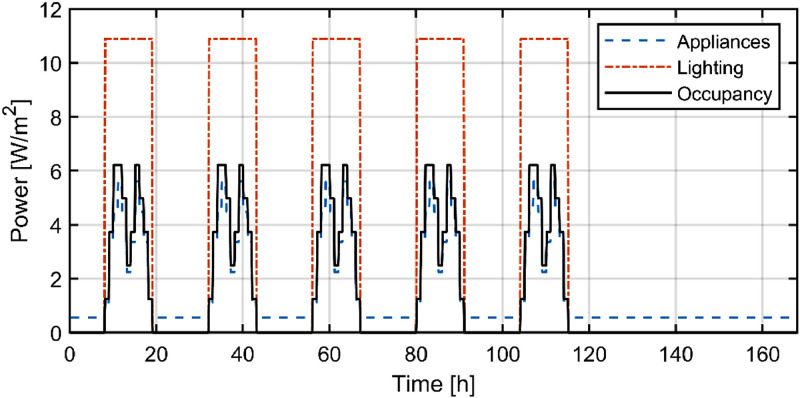
Internal gains due to appliances, lighting and occupancy [2].

Hourly schedules different for weekdays and weekends (see Fig. 6) are implemented to model a realistic user behavior (i.e. occupancy, appliances and lighting).

A more detailed description of the office cell is reported in the IEA SHC Task 56 report [1] and in Magni et al. [2].

### 2.2 Results of the building energy simulation tools

The office building used in this case study is simulated with different building energy simulation tools: EnergyPlus v.9.3, TRNSYS 18, Simulink/CarnotUIBK, Simulink/ALMABuild, IDA ICE v.4.8, Modelica Buildings library v.5.0.1 together with Dymola v. 2020x and DALEC. The different tools have different focuses and implement different models with different levels of detail. An overview of the different approaches is proposed in [2].

The analysed tools solve the numerical equations using different time steps (i.e. EnergyPlus, TRNSYS and DALEC use fixed time step while Simulink, IDA ICE and Modelica are based on variable time step) and solvers. Using a variable time step means that the solver defines the step size during the simulation, which is reduced (to increase the accuracy) when model states are changing rapidly or increased avoiding unnecessary steps when the model states are changing slowly. On the contrary, when a fixed time step is used the step size is kept constant during the whole simulation.

In all the tools the user can define the maximum time step (in case of variable time step) or the time step (in case of fixed time step) and in all the tools, except for DALEC that can only provide hourly calculations, the results are saved every 10 min. To compare the results of all the tools, hourly average powers and temperatures are calculated using the values within each hour. The resulting hourly time series (i.e. Heating power, cooling power, infiltration losses, ventilation losses, solar gains, internal gains, convective temperature, radiative temperature, ambient temperature and solar irradiation on the south façade) for each considered location (i.e. Rome, Stuttgart and Stockholm) are reported in the data file and used for the analysis of the deviations between the different tools.

It is noteworthy to mention that the solar gains can be defined differently within the different tools since they implement different window models. The hourly solar gains presented within this work represent the total solar gains including the direct transmitted solar radiation and the absorbed solar radiation which is subsequently re-emitted inside the thermal zone.

The results of the tools can be used as a reference for the cross-comparison of other office building models. In this case, the user should:

1. Create the building model starting from the description of the office cell provided in Section 2.1;
2. Run the simulation and save the results needed for the comparison with the provided benchmark;
3. If the saved results are sub-hourly, a pre-processing step is required to calculate hourly average results otherwise if the results are already on an hourly basis they can be directly used for the comparison;
4. The hourly data for the whole year can be inserted in a new column before the median column (see Table 1) and the formulas already included in the excel sheet can be used for the analysis of the deviations;
5. If the deviations are too high, the user should try to understand the possible reasons and improve the simulation model and/or inputs and repeat the sequence starting from step 2. For this step, the user could find support reading [2], where the main problems encountered during the comparison process are reported.

### 2.3 Description of the method applied for the analysis of the deviations

In the current work, not only a detailed data set of results is provided but also an approach for the evaluation of deviations between time series. A deep analysis of the challenges related to the usage of statistical indices is provided in [2] and the equations used are reported also in this section.

Since no measured data are available for this case study, it is necessary to define a set of reference data against which the results of each tool can be compared. For this purpose, the median value of the results of all the tools for each hour is calculated and used as a reference.

Both non-normalized and normalized statistical indices are calculated for the analysis of the deviations and the applied equations are reported in Table 5, where:

• $r_{i}$ represents the reference value for the i^th^ time step, calculated as the median of the results of all the tools in each considered time step;
• $s_{i}$ is the simulated value for a particular tool at the i^th^ time step;
• N is the number of considered data (i.e. corresponding to the number of time steps);
• $\bar{r}$ is the average of the reference values r;
• nm is a normalization means.

**Table 5:** Non-normalized (Mean bias error, Mean absolute error, Root mean square error) and normalized statistical indices (Normalized Mean bias error, Normalized mean absolute error, Normalized root mean square error, Coefficient of determination) [2].

Non-normalized indices	Normalized Indices
(1) $\text{MBE}=\frac{\sum_{i=1}^{N}\left(s_{i}-r_{i}\right)}{N}$	(2) $\text{NMBE}=\frac{\sum_{i=1}^{N}\left(s_{i}-r_{i}\right)}{\sum_{i=1}^{N}r_{i}}\;\left[\%\right]$
(3) $\text{MAE}=\frac{\sum_{i=1}^{N}\left|s_{i}-r_{i}\right|}{N}$	(4) $\text{NMAE}=\frac{\sum_{i=1}^{N}\left|s_{i}-r_{i}\right|}{\left|\sum_{i=1}^{N}r_{i}\right|}\;\left[\%\right]$
(5) $\text{RMSE}=\sqrt{\frac{\sum_{i=1}^{N}{\left(s_{i}-r_{i}\right)}^{2}}{N}}$	(6) $\text{NRMSE}=\frac{1}{\left|\text{nm}\right|}\sqrt{\frac{\sum_{i=1}^{N}{\left(s_{i}-r_{i}\right)}^{2}}{N}}$
	(7) $$R^{2}=1-\frac{\sum_{i=1}^{N}{\left(s_{i}-r_{i}\right)}^{2}}{\sum_{i=1}^{N}{\left(r_{i}-\bar{r}\right)}^{2}}$$

Two different normalization means (nm) are considered: the average of the reference values (see Eq. (8)) and the average of the reference values counting only the absolute values of the reference data higher than zero (see Eq. (9)).

(8) $$\text{av}=\frac{\sum_{i=1}^{N}r_{i}}{N}$$

(9) $$av_{>0}=\frac{\sum_{i=1}^{N}r_{i}}{N_{\left|r\right|>0}}$$

As highlighted in [2], normalization issues related to the average value trending to zero can be avoided using the av>0 as normalization means. This problem is particularly relevant when the variant under analysis is often close to zero (e.g. heating and cooling powers).

The normalized indices are needed to compare the calculated deviations against given thresholds (e.g. ASHRAE Guideline 14–2014 [3]) or for the comparison of the deviations between different data sets. ASHRAE Guideline 14–2014 [3] describes a method for the validation of the building model against measurements and suggests that the calculated deviations should remain below the following limits: ±5% for the monthly NMBE, 15% for the monthly NRMSE, ± 10% for the hourly NMBE, 30% for the hourly NRMSE and > 0.75 for the R2.

It is noteworthy to mention that the RMSE is scale-dependent and can be calculated only for data based on a scale with an absolute zero (e.g. Kelvin for temperatures).

The spreadsheet presented in the current work can be used as a reference for the validation of other models of office cell as the one described in this work. In addition, the statistical evaluation included in the spreadsheet can also be used for the comparison of simulation results against measurement data. In this case, the measurement data should replace the median as a reference and the simulated results should overwrite the results of the tools.

## CRediT authorship contribution statement

**Mara Magni:** Conceptualization, Methodology, Software, Validation, Formal analysis, Investigation, Data curation, Writing – original draft, Visualization. **Fabian Ochs:** Supervision, Project administration, Funding acquisition, Writing – review & editing. **Samuel de Vries:** Software, Validation. **Alessandro Maccarini:** Software. **Ferdinand Sigg:** Software.

## Declaration of Competing Interest

The authors declare that they have no known competing financial interests or personal relationships which have or could be perceived to have influenced the work reported in this article.

## Appendix Supplementary materials

Image, application 1
